# ICG enhances the effectiveness of lymphatic dissection by improving surgical performance

**DOI:** 10.1097/JS9.0000000000001658

**Published:** 2024-05-20

**Authors:** Hongrui Chen, Bin Sun, Chen Hua, Xiaoxi Lin

**Affiliations:** a Department of Plastic and Reconstructive Surgery, Shanghai Ninth People’s Hospital, affiliated to Shanghai Jiao Tong University School of Medicine, Shanghai, People’s Republic of China

*Dear Editor,*

We have thoroughly examined the article entitled ‘OSATS scoring confirms ICG enhancement of performance in laparoscopic radical gastrectomy: a post-hoc analysis of a randomized controlled trial,’ authored by Huang *et al*.^1^, published in the *International Journal of Surgery*. This research offers valuable insights into the reasons behind the increased number of lymph node dissections facilitated by Indocyanine Green (ICG) fluorescence imaging in laparoscopic radical gastrectomy, guiding the application of ICG in this critical field. Although the study is comprehensive, we believe there are significant aspects worth further discussion.

While the study demonstrated that the application of ICG fluorescence navigation technology increased OSATS (Objective Structured Assessments of Technical Skills) scores and reduced the rate of non-compliance in lymph node dissection, the exclusive use of the OSATS system for surgical process scoring might lean toward subjectivity. Moreover, the differences in surgical approaches between the two groups influenced the OSATS scoring of surgery videos: the operations in the ICG group were more distinctly identifiable; thus, the assessment and scoring lacked objectivity. Hence, incorporating other scoring systems for further analysis might enhance the credibility of the research^2^.

In the study, there were significant differences in baseline characteristics between the ICG and non-ICG groups; a larger proportion of proximal tumor patients in the non-ICG group (67%) required total gastrectomy and Roux-en-Y reconstruction compared to the ICG group (45%). Patients undergoing total gastrectomy (TG) scored significantly lower in OSATS compared to distal gastrectomy (DG), possibly due to the higher technical demands of TG. Patients receiving ICG injections had more distal tumors and underwent DG, resulting in higher OSATS scores for them. Therefore, further stratified analysis of the total gastrectomy and distal gastrectomy groups should be conducted to eliminate the impact of surgical technical difficulty on the study outcomes, thus enhancing the reader’s understanding.

The authors indicated that the application of ICG technology can increase the number of lymph nodes dissected and defined an increase in the number of resected lymph nodes (RLNs) as RLNs ≥40^3^. This statement, based on research from Japan, suggests that RLNs ≥40 is an independent prognostic factor for stage III gastric cancer patients after total gastrectomy, with patients in the RLNs ≥40 group having better prognoses than those with RLNs <40. My concern is that this study only evaluated stage III gastric cancer patients undergoing total gastrectomy without analyzing patients of other stages and surgical methods. The authors’ use of RLNs ≥40 as a basis for increased lymph node dissection may lack credibility, and it is recommended to employ appropriate statistical methods to confirm RLNs =40 as the optimal cutoff value for lymph node numbers.

Although Huang and colleagues’ research marked an important step in understanding the application of ICG in gastric cancer, addressing these issues could significantly strengthen the study’s outcomes and applicability. A broad discussion of these matters will greatly benefit the surgical and medical community, ensuring that future research is not only methodologically sound but also aligns with evolving clinical needs and realities. We commend the authors for their significant contribution to the field and look forward to further discussions to enhance our collective understanding.

## Ethical approval

Ethical approval was not required.

## Consent

Not applicable.

## Sources of funding

This research was supported by the Major and Key Cultivation Projects of Ninth People’s Hospital affiliated with Shanghai Jiao Tong University School of Medicine (No. JYZP005) and the Fundamental Research Funds for the Central Universities (No. YG2023ZD13).

## Author contribution

H.C., B.S., C.H., and X.L.: designed and wrote this letter.

## Conflicts of interest disclosure

There are no conflicts of interest.

## Research registration unique identifying number (UIN)

This study has no human subjects.

1. Registry used: not applicable.
2. Unique identifying number or registration ID: not applicable.
3. Hyperlink to your specific registration (must be publicly accessible and will be checked): not applicable.

## Guarantor

Xiaoxi Lin.

## Data availability statement

Not applicable.

## Provenance and peer review

Not commissioned, externally peer-reviewed.
